# Prevalence and factors associated with neonatal hypoglycemia in Northern Uganda: a community-based cross-sectional study

**DOI:** 10.1186/s41182-020-00275-y

**Published:** 2020-11-04

**Authors:** David Mukunya, Beatrice Odongkara, Thereza Piloya, Victoria Nankabirwa, Vincentina Achora, Charles Batte, James Ditai, Thorkild Tylleskar, Grace Ndeezi, Sarah Kiguli, James K. Tumwine

**Affiliations:** 1 Sanyu Africa Research Institute, Mbale, Uganda; 2 Center for Intervention Science in Maternal and Child Health (CISMAC), Center for International Health, University of Bergen, Bergen, Norway; 3 Busitema University Faculty of Health Sciences, Mbale, Uganda; 4 Department of Pediatrics, University of Gulu, Gulu, Uganda; 5 Center for International Health, University of Bergen, Bergen, Norway; 6 Department of Pediatrics and Child Health, Makerere University, Kampala, Uganda; 7 Department of Epidemiology and Biostatistics, School of Public Health, Makerere University, Kampala, Uganda; 8 Department of Obstetrics and Gynecology, University of Gulu, Gulu, Uganda; 9 Lung Institute, Makerere University, Kampala, Uganda

**Keywords:** Hypoglycemia, Newborn care, Breastfeeding, Neonatal care, Endocrinology

## Abstract

**Background:**

Neonatal hypoglycemia is the most common endocrine abnormality in children, which is associated with increased morbidity and mortality. The burden and risk factors of neonatal hypoglycemia in rural communities in sub-Saharan Africa are unknown.

**Objective:**

To determine the prevalence and risk factors for neonatal hypoglycemia in Lira District, Northern Uganda.

**Methods:**

This was a community-based cross-sectional study, nested in a cluster randomized controlled trial designed to promote health facility births and newborn care practices in Lira District, Northern Uganda. This study recruited neonates born to mothers in the parent study. Random blood glucose was measured using an On Call® Plus glucometer (ACON Laboratories, Inc., 10125 Mesa Road, San Diego, CA, USA). We defined hypoglycemia as a blood glucose of < 47 mg/dl. To determine the factors associated with neonatal hypoglycemia, a multivariable linear regression mixed-effects model was used.

**Results:**

We examined 1416 participants of mean age 3.1 days (standard deviation (SD) 2.1) and mean weight of 3.2 kg (SD 0.5). The mean neonatal blood glucose level was 81.6 mg/dl (SD 16.8). The prevalence of a blood glucose concentration of < 47 mg/dl was 2.2% (31/1416): 95% CI 1.2%, 3.9%. The risk factors for neonatal hypoglycemia were delayed breastfeeding initiation [adjusted mean difference, − 2.6; 95% CI, − 4.4, − 0.79] and child age of 3 days or less [adjusted mean, − 12.2; 95% CI, − 14.0, − 10.4].

**Conclusion:**

The incidence of neonatal hypoglycemia was low in this community and was predicted by delay in initiating breastfeeding and a child age of 3 days or less. We therefore suggest targeted screening and management of neonatal hypoglycemia among neonates before 3 days of age and those who are delayed in the onset of breastfeeding.

## Introduction

Neonatal hypoglycemia, defined differently by various authors as random blood sugars ranging from 18 to 72 mg/dl [1–3], is the most common metabolic abnormality in newborns and results in increased morbidity and mortality [1, 4, 5]. The risk of neonatal hypoglycemia is particularly high in preterms, low birth weight neonates, and neonates born to diabetic mothers [1, 6]. Ironically, neonates commonly develop transient hypoglycemia in the first few hours of life as a normal physiological process [3, 7]. However, some neonates progress to a severe and prolonged form of neonatal hypoglycemia, which can result in seizures and poor neuro-developmental outcomes if poorly managed [1, 3].

Currently, there is no consensus on the appropriate glucose cutoff value that differentiates transient hypoglycemia from the prolonged pathological form of neonatal hypoglycemia [3]. Various authors have suggested cutoff levels ranging from 47 to 60 mg/dl [2, 3]. The proposed cutoffs may not be applicable to newborns in sub-Saharan Africa that are breastfed early, and for longer periods [8]. Moreover, practices such as immediate umbilical cord clamping [9] and home births are common in some parts of sub-Saharan Africa [10], which may result in differing incidence and outcomes of neonatal hypoglycemia. Whereas transient neonatal hypoglycemia in the first 48 h is often inconsequential [3, 7], there is some evidence that a single episode of transient hypoglycemia may result in neuro-developmental abnormalities [11].

Although universal screening of asymptomatic and low-risk neonates for hypoglycemia may be unnecessary and harmful [3, 12], there is evidence that asymptomatic hypoglycemia could result in neuro-developmental abnormalities in up to 20% of affected neonates [13, 14]. Moreover, context-specific risk factors in rural communities in sub-Saharan Africa that could guide screening are unknown.

We therefore aimed to determine the incidence and risk factors of neonatal hypoglycemia in the first 7 days of life in a rural community in Northern Uganda to enable development of contextually relevant screening guidelines for neonatal hypoglycemia.

## Materials and methods

### Study design

This was a community-based cross-sectional study of neonates born to women enrolled in a cluster randomized controlled trial evaluating the effect of peer counseling on health facility births (Survival Pluss study registered on ClinicalTrial.gov as NCT02605369).

### Study setting

The study was conducted in Lira District, Northern Uganda, between January 2018 and March 2019. Lira District is approximately 340 km from the capital city, Kampala, and has 13 sub-counties, 1 municipality, and 751 villages. We recruited from Aromo, Agweng, and Ogur sub-counties located in the northern part of the district. These sub-counties were chosen because they were to be the site of the parent study. At the time of the study, the population of Lira District is ~ 400,000 people. The majority of the people lived in rural areas and practice subsistence farming [15]. The Uganda Demographic and Health Survey conducted in 2016 reported that ~ 29 of every 1000 newborns died in the first 28 days of life in the region covering the Lira District [16].

### Survival Pluss study (the parent study)

The parent study was a community-based cluster randomized controlled trial designed to evaluate the effect of a combined intervention on the proportion of mothers giving birth in health facilities. The combined intervention consisted of peer counseling, mobile phone messaging, and distribution of mama kits. The unit of randomization was a cluster, made up of 5 to 10 villages with a population of > 1000 people. Up to 30 clusters were randomized (ratio of 1:1) to the intervention or control arm. Mothers were enrolled in the third trimester of pregnancy and followed up for 50 days postpartum. Each village had a recruiter (pregnancy monitor) who was elected during a community meeting and who notified the research team of all pregnant women in her village during the study period and of all births. During home visits, research assistants recruited women of 28 or more weeks pregnant who were resident in the selected clusters. They were followed up on days 1, 7, 28, and 52 postpartum.

### Study participants

All newborns of mothers participating in the cluster randomized controlled trial, who were alive on the day of examination, within 1 week of birth, and whose guardians consented to a glucose measurement, were eligible for the study. Severely ill neonates who were admitted to hospitals at the time of the study were excluded.

### Study procedure

Trained midwives visited the mother as soon after birth as possible, but no later than 1 week after birth, and obtained a random blood sugar by pricking the newborn’s heel. Random blood glucose was measured in mmol/l using an On Call® Plus glucometer (ACON Laboratories, Inc., 10125 Mesa Road, San Diego, CA, USA), a point-of-care test. Under aseptic conditions, we obtained blood samples from the heels of neonates. The heel was first cleaned with alcohol swabs and dried with cotton. A single-use safety lancet was used to prick the heel. Maternal random blood glucose was also obtained at the same time from a finger prick. The team was closely supervised by a pediatric endocrinologist and a medical doctor who had trained them on sample collection, observed their initial procedures, and occasionally sitting in during the recruitment visits to ensure the standard operating procedures were followed.

### Study variables

To determine risk factors for neonatal hypoglycemia, we analyzed neonatal blood glucose as a continuous outcome. To determine short-term outcomes of neonatal hypoglycemia, we used a categorized neonatal blood glucose measurement. A cutoff of < 47 mg/dl was used, as it was most commonly used in prior studies and is not that different from more recent suggestions [2, 3, 17–19]. We, however, also investigated cutoffs of < 60 mg/dl and < 70 mg/dl [3]. Data was collected on several risk factors during pregnancy and immediately after birth. This included maternal age, parity, maternal education, paternal education, wealth, singleton or multiple birth, sex of the newborn, place of birth, birth weight, early breastfeeding initiation, bathing of the newborn, maternal BMI, age of baby, and the place the newborn was immediately after birth. Wealth quintiles were calculated from an asset-based index using principal component analysis [20], based on ownership of assets in the household, including mobile phone, radio, land, cupboard, bicycle, motorcycle, and assessing the household dwelling characteristics—material of the floor, roof, and wall. We defined early breastfeeding initiation as the initiation of breastfeeding within 1 h of birth and delayed breastfeeding initiation as the initiation of breastfeeding later than 1 h after birth. Low birth weight was defined as being < 2.5 kg.

### Power and sample size

The sample size was limited by the size of the parent study. We enrolled 1416 neonates who were part of the parent cluster randomized trial. This sample size results in an absolute precision of 1.2 to 4.4%, i.e., the difference between the point estimate and the 95% confidence interval (CI) for incidence values ranging from 2 to 50%.

### Data analysis

We summarized categorical variables as proportions and continuous variables as means (SD) or medians (IQR) and compared them using Student’s *t* tests or Mann-Whitney *U* tests as appropriate. The prevalence of neonatal hypoglycemia was defined as blood glucose < 47 mg/dl. We used linearized variance estimation adjusting for clustering to compute the confidence intervals around the estimates. To determine the factors associated with neonatal hypoglycemia, a multivariable linear regression mixed-effects model was used in which the random effect was the cluster. Based on scientific literature and biological plausibility, the following covariates were added to the fixed effects part of the model, low birth weight, delayed breastfeeding initiation, bathing of the baby in the first 24 h, maternal hyperglycemia (blood glucose ≥ 198 mg/dl), any maternal complication during birth, maternal age, maternal education, parity, place of birth, wealth index, and cesarean section. Since this study was nested in a cluster randomized controlled trial, the trial arm was added as a fixed effect. We assumed an exchangeable correlation and used maximum likelihood estimation in fitting the model. All analyses were done using STATA 14.0 (StataCorp, College Station, TX, USA).

### Ethical considerations

Ethical approval for the study was obtained from the following bodies: (1) Research and Ethics committee School of Medicine, Makerere University (SOMREC: REF 2015-121); (2) Uganda National Council of Science and Technology (UNCST: SS 3954); and (3) Regional Committees for Medical and Health Research Ethics (REK VEST 2017/2079). We obtained written informed consent from the caretakers of all participants in the study. Participants whose neonates were hypoglycemic were encouraged to breastfeed immediately and, when necessary, a referral to the nearest health facility was facilitated.

## Results

### Participant characteristics

**Fig 1:**
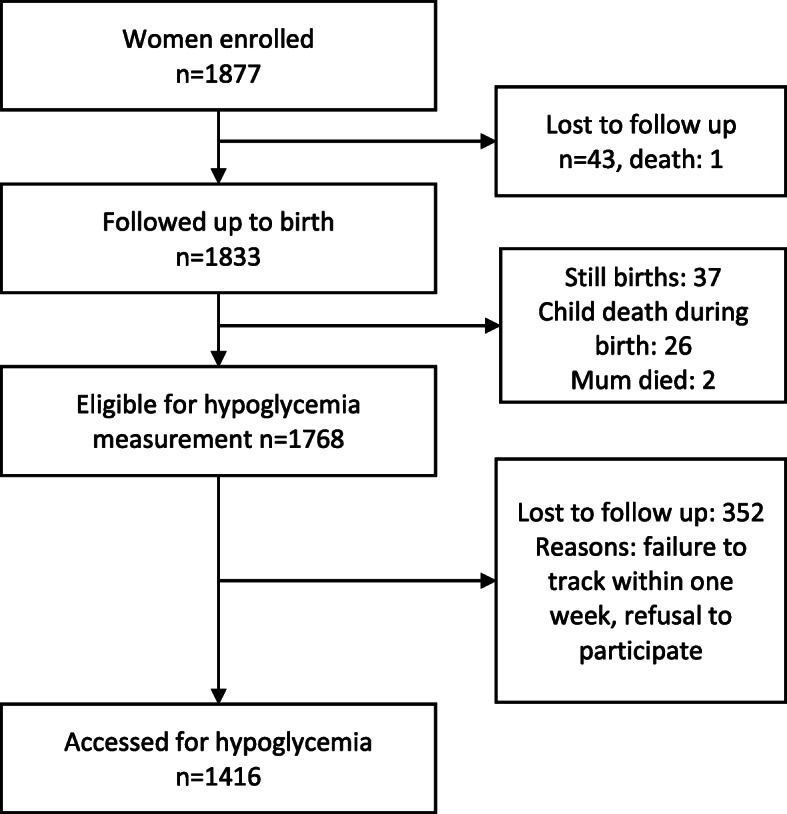
Study profile of neonates assessed for hypoglycemia in Lira District, Northern Uganda

We examined 1416 participants (Fig. 1). The mean age of participants was 3.1 days (standard deviation (SD) 2.1). The mean weight of the participants was 3.2 kg (SD 0.5). The average age of their mothers was 24.7 years (6.8). Further characteristics are given in Table 1.

**Table 1:** Characteristics of newborns assessed for hypoglycemia in Northern Uganda

Variable	Frequency (*n* = 1416)	Percentage
**Mother’s age**		
≤ 19	369	26.1
20-30	760	53.7
> 30	287	20.3
**Mother’s education**		
None	184	13
Primary	1107	78.2
Secondary	107	7.6
Tertiary	18	1.3
**Father’s education**		
None	25	1.8
Primary	843	59.5
Secondary	347	24.5
Tertiary	77	5.4
Missing	124	8.8
**Parity**		
≤ 1	637	45
2-4	484	34.2
> 4	295	20.8
**Place of birth**		
Home	464	32.8
Health facility	951	67.2
Missing	1	0.1
**Cesarean section**		
No	1380	97.5
Yes	36	2.5
**Marital status**		
Single	124	8.8
Married	1292	91.2
**Electricity**		
No	1262	89.1
Yes	154	10.9
**Delayed or no cry**		
No	1347	95.1
Yes	69	4.9
**Birth weight**		
Normal	1153	81.4
Low birth weight	75	5.3
Missing	188	13.3
**Phone in home**		
No	623	44
Yes	793	56
**Wealth index**		
Poorest	286	20.2
2	349	24.6
3	268	18.9
4	243	17.2
Richest	270	19.1
**Oxygen administered**		
No	1402	99.0
Yes	13	0.9
Missing	1	0.1
**Bathed baby in first 24 h**		
No	591	41.7
Yes	820	57.9
Missing	5	0.4
**Maternal antenatal BMI**		
< 18.5	11	0.8
18.5-24.9	1174	83.7
25-29.9	194	13.7
≥ 30	24	1.7
Missing	13	0.9
**Maternal hyperglycemia**		
No	1393	98.4
Yes	23	1.6
**Breastfeeding initiation**		
Late	530	37.4
Early	876	61.9
Missing	10	0.7

### Proportion of neonates with hypoglycemia in the first 7 days of life

The mean neonatal blood glucose level was 81.6 mg/dl (SD 16.8), and the median blood glucose 81 (IQR 70.2, 93.6). The prevalence of a blood glucose concentration < 47 mg/dl was 2.2% (31/1416): 95% CI 1.2%, 3.9%.

### Risk factors for neonatal hypoglycemia

**Fig. 2:**
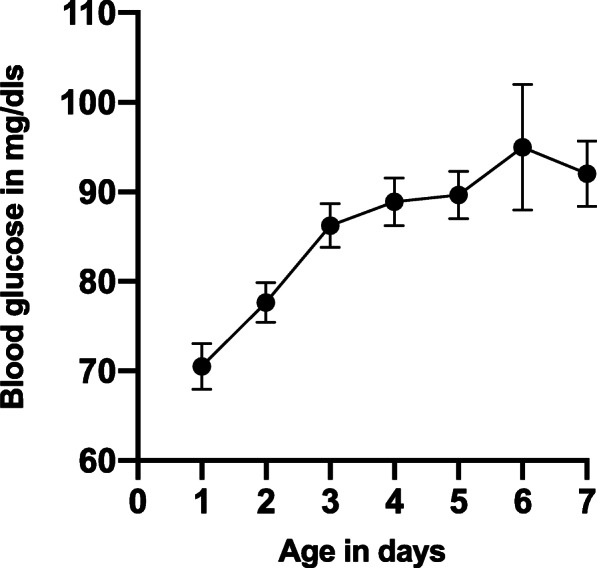
Mean blood glucose, with 95% confidence intervals, of neonates from the age of 1 to 7 days in Northern Uganda

The risk factors for neonatal hypoglycemia were delayed breastfeeding initiation, bathing the baby in the first 24 h after birth, and the baby’s age 3 days or younger at examination. Mean blood glucose levels were 2.6 mg/dl lower among neonates who were breastfed later than 1 h compared to those who were breastfed in the first hour after birth [adjusted mean difference, − 2.6; 95% CI, − 4.4, − 0.79]. Neonates bathed within the first 24 h after birth had on average 2.3 mg/dl higher glucose concentration than those who were bathed afterwards [adjusted mean 2.3; 95% CI, 0.46, 4.2]. At the time of examination, neonates 3 days old or younger had an average of 12.2 mg/dl lower glucose concentration than those over 3 days [adjusted mean, − 12.2; 95% CI, − 14.0, − 10.4] (Table 2, Fig. 2).

**Table 2:** Risk factors of neonatal hypoglycemia in Northern Uganda

	Bivariable	Multivariable
	Unadjusted mean difference (95 mg/dl% CI)	Adjusted mean difference (95% CI)
**Intervention group**		
Control	0	0
Intervention	− 1.6 (− 4.1, 0.84)	− 1.2 (− 3.4, 0.99)
**Maternal hyperglycemia**		
No	0	0
Yes	− 0.61 (− 7.5, 6.3)	− 0.22 (− 7.2, 6.7)
**Age of neonate**		
> 3 days		0
≤ 3 days	− 12.9 (− 14.5, − 11.2)	− **12.2 (**− **14.0,** − **10.4)**
**Maternal antenatal BMI**		
< 18.5	1.1 (− 8.9, 11.0)	1.1 (− 8.0, 10.2)
18.5-24.9	0	0
25-29.9	0.37 (− 2.2, 2.9)	1.7 (− 0.96, 4.3)
≥ 30	− 0.56 (− 7.3, 6.2)	− 0.37 (− 7.3, 6.6)
**Low birth weight (less than 2.5 kg)**		
No	0	0
Yes	− 0.76 (− 4.6, 3.1)	0.48 (− 3.1, 4.1)
**Bathed baby before visit**		
No	0	0
Yes	4.8 (3.0, 6.6)	**2.3 (0.46, 4.2)**
**Breastfeeding initiation**		
Early	0	0
Late	− 2.4 (− 4.2, 0.57)	− **2.6 (**− **4.4,** − **0.79)**
**Maternal complications during pregnancy**		
No	0	0
Yes	1.1 (− 0.65, 2.9)	− 1.2 (− 3.5, 1.1)
**Neonatal hypothermia**		
No	0	0
Yes	− 1.4 (− 3.8, 1.1)	− 1.2 (− 3.5, 1.1)
**Age of mother**		
≤ 19	0	0
20-30	1.6 (− 0.50, 3.7)	0.76 (− 1.3, 2.9)
> 30	0.30 (− 2.2, 2.9)	− 0.02 (− 2.8, 2.7)
**Mother’s education**		
None	0	0
Primary	1.2 (− 1.4, 3.8)	0.60 (− 2.1, 3.3)
≥ Secondary	1.7 (− 2.1, 5.5)	1.0 (− 3.0, 5.0)
**Place of birth**		
Health facility	0	0
Home	1.3 (− 0.65, 3.1)	− 0.20 (− 2.2, 1.8)
**Wealth quintiles**		
1 (poorest)	0	0
2	− 0.72 (− 3.3, 1.9)	− 0.63 (− 3.2, 2.0)
3	− 1.4 (− 4.2, 1.4)	− 1.7 (− 4.4, 1.1)
4	− 0.52 (− 2.4, 3.4)	0.11 (− 2.8, 3.0)
5 (richest)	− 0.30 (− 3.1, 2.5)	− 0.93 (− 3.8, 1.9)

## Discussion

The prevalence of neonatal hypoglycemia in the first week of life was low (2.2%). The mean random blood glucose of our sample population was 82.1 mg/dl (SD 17.5), which is much lower than reported by others [21–24], possibly for two reasons. First, our population had high levels of early breastfeeding initiation and continued breastfeeding [16]. Since breastfeeding prevents and resolves neonatal hypoglycemia [1, 3], the neonates who could or might have suffered from neonatal hypoglycemia were promptly managed. Second, the study population had a very low prevalence of maternal hyperglycemia (a marker of diabetes mellitus) and low birth weight (a marker of prematurity). This corresponds to findings from a nationwide survey in Uganda that reported a prevalence of impaired fasting glycemia of 2% [25]. Since maternal hyperglycemia is one of the causes of neonatal hypoglycemia [3], the low population prevalence could partly explain the low prevalence of it in our selected population. Nonetheless, our findings are similar to those obtained from two American and one Indian study [26–28].

Delayed breastfeeding initiation was associated with neonatal hypoglycemia. This finding is not surprising, and it has been reported by previous authors [21, 28, 29]; breastfeeding is an initial means of correcting neonatal hypoglycemia [1]. This finding reinforces the need to encourage mothers to breastfeed their babies within the first hour after birth. It also sheds light on a potential mechanism through which delayed breastfeeding could increase the risk of neonatal morbidity and mortality [30].

Bathing the newborn within 24 h after birth was also associated with neonatal hypoglycemia. This can be explained by the fact that bathing newborns within 24 h of birth predisposes them to cold stress and hypothermia [31], which are risk factors for neonatal hypoglycemia [28]. However, in our study sample, the association between hypothermia and hypoglycemia was very weak and imprecise. As such, the association between bathing the newborn within 24 h and hypoglycemia could be non-casual. This non-causal association could result from both neonatal hypoglycemia and bathing newborns within 24 h after birth causing neonatal hypothermia [32]. This would result in a conditional association between neonatal hypoglycemia and bathing the newborn within 24 h after birth. We therefore suggest that this association could result from a form of collider bias [32–35].

Neonates of 3 days or younger had lower blood glucose concentrations compared to older ones. The incidence of neonatal hypoglycemia decreases as the child ages [21], which might explain this difference. This is because physiological transitional hypoglycemia resolves within the first 48-72 h, after which blood neonatal blood glucose levels gradually increase [3, 22].

### Limitations

Our study had some limitations. First, our loss to follow-up and inability to reach some neonates within the first week of life might have resulted in selection bias. Since we did not examine hospitalized neonates, who might have had lower blood glucose values than healthier neonates, we could have underestimated the burden of neonatal hypoglycemia. Second, we could only take one blood glucose measurement, which could have resulted in a lower estimate of neonatal hypoglycemia. We recommend that future studies take repeated blood sugar measurements if possible. Finally, we did not obtain information on the last time the child was breastfed prior to blood glucose sampling, or on the consumption of products such as tea and herbs prior to our test.

## Conclusion

The incidence of neonatal hypoglycemia was low in this community and was predicted by delayed breastfeeding initiation and child age of 3 days or less. We therefore suggest targeted screening and management of neonatal hypoglycemia among neonates younger than 3 days and those who experience delay in breastfeeding initiation.

## Data Availability

The datasets used and/or analyzed during this study are available from the corresponding author on reasonable request.

## Abbreviations

SD: Standard deviation
BMI: Body mass index
CI: Confidence interval
IQR: Inter-quartile range
UNCST: Uganda National Council of Science and Technology

## References

[1] Jain A, Aggarwal R, Jeevasanker M, Agarwal R, Deorari AK, Paul VK (2008). Hypoglycemia in the newborn. Indian J Pediatr.

[2] Lucas A, Morley R, Cole TJ (1988). Adverse neurodevelopmental outcome of moderate neonatal hypoglycemia. Bmj..

[3] Thompson-Branch A, Havranek T (2017). Neonatal hypoglycemia. Pediatr Rev.

[4] Yismaw AE, Gelagay AA, Sisay MM (2019). Survival and predictors among preterm neonates admitted at University of Gondar comprehensive specialized hospital neonatal intensive care unit, Northwest Ethiopia. Ital J Pediatr.

[5] Kutamba E, Lubega S, Mugalu J, Ouma J, Mupere E (2014). Dextrose boluses versus burette dextrose infusions in prevention of hypoglycemia among preterms admitted at Mulago Hospital: an open label randomized clinical trial. Afr Health Sci.

[6] Stanescu A, Stoicescu SM. Neonatal hypoglycemia screening in newborns from diabetic mothers--arguments and controversies. J Med Life. 2014;7 Spec No. 3:51-52. PMC4391423 25870695

[7] Stomnaroska-Damcevski O, Petkovska E, Jancevska S, Danilovski D (2015). Neonatal hypoglycemia: a continuing debate in definition and management. Pril (Makedon Akad Nauk Umet Odd Med Nauki).

[8] Textor J, van der Zander B, Gilthorpe MS, Liskiewicz M, Ellison GT (2016). Robust causal inference using directed acyclic graphs: the R package ‘dagitty’. Int J Epidemiol.

[9] Senior B (1973). Neonatal hypoglycemia. N Engl J Med.

[10] Mukunya D, Tumwine JK, Ndeezi G, et al. Inequity in utilization of health care facilities during childbirth: a community-based survey in post-conflict Northern Uganda. J Public Health (Berl). 2019:1–9. 10.1007/s10389-019-01114-z.

[11] Kaiser JR, Bai S, Gibson N (2015). Association between transient newborn hypoglycemia and fourth-grade achievement test proficiency: a population-based study. JAMA Pediatr.

[12] Haninger NC, Farley CL (2001). Screening for hypoglycemia in healthy term neonates: effects on breastfeeding. J Midwifery Womens Health.

[13] Singhal PK, Singh M, Paul VK, Deorari AK, Ghorpade MG, Malhotra A (1992). Neonatal hypoglycemia--clinical profile and glucose requirements. Indian Pediatr.

[14] Williams AF (1997). Hypoglycemia of the newborn: a review. Bull World Health Organ.

[15] Uganda Bureau of Statistics. The National Population and Housing Census 2014-Main Report. Kampala, Uganda: 2016. https://www.dhsprogram.com/pubs/pdf/FR333/FR333.pdf.

[16] Uganda Bureau of Statistics (UBOS) and ICF (2018). Uganda Demographic and Health Survey 2016.

[17] McKinlay CJ, Alsweiler JM, Ansell JM (2015). Neonatal glycemia and neurodevelopmental outcomes at 2 years. N Engl J Med.

[18] Alkalay AL, Sarnat HB, Flores-Sarnat L, Elashoff JD, Farber SJ, Simmons CF (2006). Population meta-analysis of low plasma glucose thresholds in full-term normal newborns. Am J Perinatol.

[19] Shah R, Harding J, Brown J, McKinlay C (2019). Neonatal glycaemia and neurodevelopmental outcomes: a systematic review and meta-analysis. Neonatology..

[20] Rutstein SO, Johnson K (2004). The DHS wealth index. DHS comparative reports no. 6.

[21] Samayam P, Ranganathan PK, Kotari UD, Balasundaram R (2015). Study of asymptomatic hypoglycemia in full term exclusively breastfed neonates in first 48 hours of life. J Clin Diagn Res.

[22] Heck LJ, Erenberg A (1987). Serum glucose levels in term neonates during the first 48 hours of life. J Pediatr.

[23] Bromiker R, Perry A, Kasirer Y, Einav S, Klinger G, Levy-Khademi F (2019). Early neonatal hypoglycemia: incidence of and risk factors. A cohort study using universal point of care screening. J Matern Fetal Neonatal Med..

[24] Cole MD, Peevy K (1994). Hypoglycemia in normal neonates appropriate for gestational age. J Perinatol.

[25] Bahendeka S, Wesonga R, Mutungi G, Muwonge J, Neema S, Guwatudde D (2016). Prevalence and correlates of diabetes mellitus in Uganda: a population-based national survey. Trop Med Int Health.

[26] Ogunyemi D, Friedman P, Betcher K (2017). Obstetrical correlates and perinatal consequences of neonatal hypoglycemia in term infants. J Matern Fetal Neonatal Med.

[27] DePuy AM, Coassolo KM, Som DA, Smulian JC (2009). Neonatal hypoglycemia in term, nondiabetic pregnancies. Am J Obstet Gynecol.

[28] Sasidharan CK, Gokul E, Sabitha S (2004). Incidence and risk factors for neonatal hypoglycemia in Kerala, India. Ceylon Med J.

[29] De AK, Biswas R, Samanta M, Kundu CK (2011). Study of blood glucose level in normal and low birth weight newborns and impact of early breast feeding in a tertiary care centre. Annals of Nigerian Medicine.

[30] Neovita Study Group (2016). Timing of initiation, patterns of breastfeeding, and infant survival: prospective analysis of pooled data from three randomised trials. Lancet Glob Health.

[31] Tasew H, Gebrekristos K, Kidanu K, Mariye T, Teklay G (2018). Determinants of hypothermia on neonates admitted to the intensive care unit of public hospitals of Central Zone, Tigray, Ethiopia 2017: unmatched case-control study. BMC Res Notes.

[32] Hernan MA, Hernandez-Diaz S, Robins JM (2004). A structural approach to selection bias. Epidemiology..

[33] Greenland S, Pearl J, Robins JM (1999). Causal diagrams for epidemiologic research. Epidemiology..

[34] Ananth CV, Schisterman EF (2017). Confounding, causality, and confusion: the role of intermediate variables in interpreting observational studies in obstetrics. Am J Obstet Gynecol.

[35] Shrier I, Platt RW (2008). Reducing bias through directed acyclic graphs. BMC Med Res Methodol.

